# Improved Powdery Mildew Resistance of Transgenic *Nicotiana benthamiana* Overexpressing the *Cucurbita moschata CmSGT1* Gene

**DOI:** 10.3389/fpls.2019.00955

**Published:** 2019-07-25

**Authors:** Wei-Li Guo, Bi-Hua Chen, Yan-Yan Guo, He-Lian Yang, Jin-Yan Mu, Yan-Li Wang, Xin-Zheng Li, Jun-Guo Zhou

**Affiliations:** ^1^School of Horticulture Landscape Architecture, Henan Institute of Science and Technology, Xinxiang, China; ^2^Henan Province Engineering Research Center of Horticultural Plant Resource Utilization and Germplasm Enhancement, Xinxiang, China

**Keywords:** *Cucurbita moschata* Duch., powdery mildew, *CmSGT1*, functional analysis, *N. benthamiana*

## Abstract

Powdery mildew (PM), which is mainly caused by *Podosphaera xanthii*, is a serious biotrophic pathogen disease affecting field-grown and greenhouse-grown cucurbit crops worldwide. Because fungicides poorly control PM, the development and cultivation of PM-resistant varieties is critical. A homolog of *SGT1* (*suppressor of the G2 allele of skp1*), which encodes a key component of the plant disease-associated signal transduction pathway, was previously identified through a transcriptomic analysis of a PM-resistant pumpkin (*Cucurbita moschata*) inbred line infected with PM. In this study, we have characterized this *SGT1* homolog in *C. moschata*, and investigated its effects on biotic stress resistance. Subcellular localization results revealed that CmSGT1 is present in the nucleus. Additionally, *CmSGT1* expression levels in the PM-resistant material was strongly induced by PM, salicylic acid (SA) and hydrogen peroxide (H_2_O_2_). In contrast, SA and H_2_O_2_ downregulated *CmSGT1* expression in the PM-susceptible material. The ethephon (Eth) and methyl jasmonate (MeJA) treatments upregulated *CmSGT1* expression in both plant materials. The constitutive overexpression of *CmSGT1* in *Nicotiana benthamiana* (*N. benthamiana*) minimized the PM symptoms on the leaves of PM-infected seedlings, accelerated the onset of cell necrosis, and enhanced the accumulation of H_2_O_2_. Furthermore, the expression levels of *PR1a* and *PR5*, which are SA signaling transduction markers, were higher in the transgenic plants than in wild-type plants. Thus, the transgenic *N. benthamiana* plants were significantly more resistant to *Erysiphe cichoracearum* than the wild-type plants. This increased resistance was correlated with cell death, H_2_O_2_ accumulation, and upregulated expression of SA-dependent defense genes. However, the chlorosis and yellowing of plant materials and the concentration of bacteria at infection sites were greater in the transgenic *N. benthamiana* plants than in the wild-type plants in response to infections by the pathogens responsible for bacterial wilt and scab. Therefore, *CmSGT1*-overexpressing *N. benthamiana* plants were hypersensitive to these two diseases. The results of this study may represent valuable genetic information for the breeding of disease-resistant pumpkin varieties, and may also help to reveal the molecular mechanism underlying CmSGT1 functions.

## Introduction

The genus Cucurbita is composed of several species, including the cultivated *Cucurbita moschata* Duch., *Cucurbita pepo* L., *Cucurbita maxima* Duch., and several wild species. Pumpkins (*C. moschata*) are valued for their fruit and seeds. Additionally, they are rich in vitamins, amino acids, flavonoids, phenolics, and carbohydrates, and possess medicinal properties, including anti-diabetic, anti-oxidant, anti-carcinogenic, and anti-inflammatory activities (Wang et al., 2002; Yadav et al., 2010). In China alone, the annual yield of pumpkin, squash, and gourds is 8,051,495 t (i.e., approximately 22.68% of the global yield) from a harvested area of 438,466 ha (i.e., 17.42% of the global area) (Food and Agriculture Organization, 2017)^1^. Cucurbit powdery mildew (PM) is a serious disease affecting field-grown and greenhouse-grown cucurbit crops worldwide. The disease is mainly caused by *Podosphaera xanthii* (formerly known as *Sphaerotheca fuliginea*), which is a biotrophic plant pathogen (Perez-Garcia et al., 2009; Fukino et al., 2013). Fungicide applications poorly control PM and the long-term use of pesticides may lead to increased environmental pollution and the residual chemicals on food crops may be harmful for humans and animals. Therefore, studying the mechanism underlying PM resistance and exploiting the resistance genes to breed resistant varieties represents an effective way to control PM in pumpkin.

To date, there has been relatively little research on pumpkin (2*n* = 2*x* = 40), especially at the molecular level, which has seriously hindered developments in the fields of molecular biology and genetics. We previously conducted a RNA sequencing analysis of pumpkin inbred line highly resistant to PM (inbred line “112-2”) and identified 4,716 differentially expressed genes, including genes encoding broad-spectrum disease-resistance/susceptibility proteins [PR protein, ubiquitin, heat shock protein and Mildew Locus O (MLO)], reactive oxygen scavengers (peroxidase, superoxide dismutase, and catalase), signal transduction molecules serine/threonine protein kinase, and transcription factors (WRKY, TGA, and MYB). Additionally, candidate disease-resistance genes, such as *WRKY21*, *MLO3*, and *SGT1*, were identified (Guo et al., 2018). One of the genes cloned from the transcriptome was a homolog of *Cucumis melo SGT1* (*suppressor of the G2 allele of skp1*). The expression of this *SGT1* homolog was highly upregulated in PM-resistant material at 6 h after an inoculation with the PM fungus, but not in PM-susceptible material. However, pumpkin *SGT1* has not been functionally characterized regarding its potential involvement in the defense response to PM.

The SGT1 protein was originally defined in yeast, in which it interacts with SKP1, which is a component of the Skp1/CDC/F-box protein E3 ubiquitin ligase complex (Kitagawa et al., 1999). The SGT1 protein contains three functional domains, namely the tetratricopeptide repeat (TPR), CHORD SGT1 (CS), and the SGT1-specific sequence (SGS) (Kumar and Kirti, 2015). SGT1 is closely related to the disease resistance mediated by the plant resistance (*R*) genes. The silencing or mutation of *NbSGT1* from *Nicotiana benthamiana* (*N. benthamiana*) can lead to reduction of steady-state levels of R proteins and the loss of the mediated resistance, and *AtSGT1a* overexpression can contribute positively to resistance triggered by the NB-LRR type R proteins, and can complement for loss of *AtSGT1b* in auxin signaling (Muskett and Parker, 2003; Azevedo et al., 2006). A previous study revealed that *NbSGT1* overexpression in *N. benthamiana* accelerates the development of the hypersensitive response (HR) during *R*-mediated disease resistance (Wang et al., 2010). Additionally, *Hv-SGT1* overexpression in wheat enhances the resistance to PM, which is correlated with increased levels of reactive oxygen intermediates at the pathogen entry sites (Xing et al., 2013). Furthermore, PsoSGT1 from *Prunus sogdiana* appears to interact with molecular chaperones (RAR1 and HSP90) to activate a nucleotide-binding domain and leucine-rich repeat-containing (NB-LRR)-type protein that confers disease resistance (Zhu et al., 2017). The regulation of NB-LRR-type protein stability or substrate degradation to maintain the balance between the activation and inhibition of plant defense responses (Meldau et al., 2011; Hoser et al., 2013), contributes to the RXLR elicitor-induced HR-related cell necrosis (Xiang et al., 2017).

The recent release of the pumpkin genome provided an opportunity for an additional screening for disease-resistance genes (Sun et al., 2017). In this study, on the basis of the above-mentioned transcriptome analysis that identified differentially expressed genes responsive to PM, we functionally characterized the pumpkin homolog of *SGT1* (designated as *CmSGT1*). The transcription of *CmSGT1* in the PM-resistant inbred line “112-2” was strongly induced by PM, salicylic acid (SA), hydrogen peroxide (H_2_O_2_), ethephon (Eth), and methyl jasmonate (MeJA). Transgenic *N. benthamiana* plants that constitutively overexpressed *CmSGT1* exhibited increased resistance to PM and were hypersensitive to bacterial wilt and scab.

## Results

### Cloning of *CmSGT1* and Subcellular Localization the Encoded Protein

Pumpkin PM-related candidate genes identified in a transcriptome were reported previously (Guo et al., 2018). One of the isolated clones exhibited 89% identity at the nucleotide level to *C. melo SGT1*. A full-length clone of this homolog was obtained, and the gene was named *CmSGT1* and submitted to GenBank (accession number MH105820). The *CmSGT1* gene comprised 1,206 bp, which included a 1,080-bp open reading frame (ORF) encoding 360 amino acids. The predicted polypeptide was basic, with a pI of 5.32, and a molecular mass of 40.3 kDa. An alignment of the deduced CmSGT1 amino acid sequence with homologous sequences is presented in Supplementary Figure S1. At the amino acid level, CmSGT1 was highly similar to the SGT1 from the following plant species: *Cucumis melo* (CmSGT1, 84.4% identity), and *Cucumis sativus* (CsSGT1, 82.5% identity), *N. benthamiana* (NbSGT1.1 and NbSGT1.2, 89.5% identity), and *Arabidopsis thaliana* (AtSGT1a and AtSGT1b, 75.2% identity). The CmSGT1 sequence contained three conserved domains, namely TPR, CS, and SGS.

**FIGURE 1:**
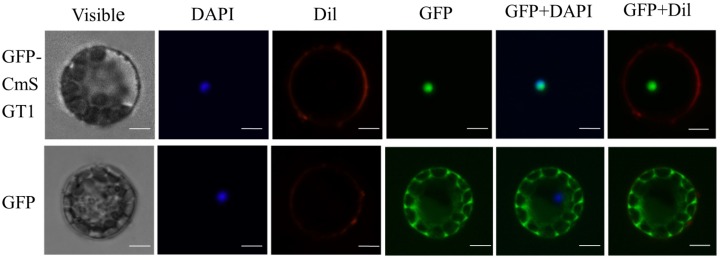
The subcellular localization of pumpkin CmSGT1. The fused pBI221-GFP- CmSGT1 and pBI221-GFP constructs were introduced into *Arabidopsis* protoplast by polyethylene glycol (PEG)-mediated protoplast transformation. The fluorescent signals were detected using a confocal fluorescence microscope. Scale bars = 5 μm.

The subcellular localization of CmSGT1 was assessed with a CmSGT1-GFP fusion protein that was produced in *A. thaliana* protoplasts under the control of the 35S CaMV promoter. The GFP signal in protoplasts producing GFP alone was detected in the cytoplasm and nucleus (Figure 1), whereas the signal from the CmSGT1-GFP fusion protein was detected exclusively in the nucleus.

### Expression of *CmSGT1* in Pumpkin Seedlings in Response to PM and Exogenous Treatments

**FIGURE 2:**
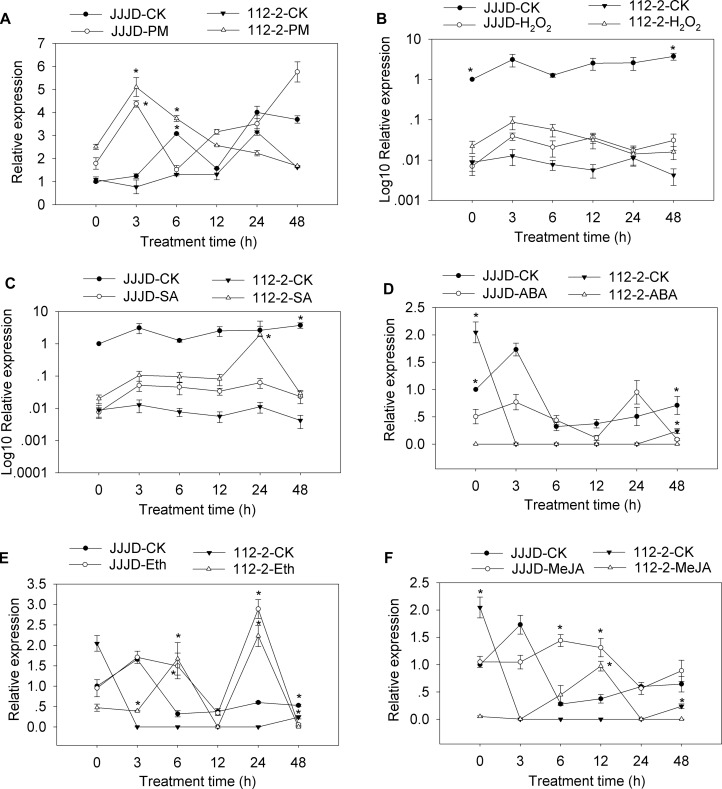
*CmSGT1* expression in response to powdery mildew and exogenous hormones. The pumpkin seedlings were sprayed with a spore suspension **(A)**, exogenous H_2_O_2_ **(B)**, SA **(C)**, ABA **(D)**, Eth **(E)** and MeJA **(F)**. The pumpkin β-*actin* gene was used as an internal reference gene for qRT-PCR. The transcript level of *CmSGT1* in the susceptible cultivar “JJJD” at 0 h is used as control (quantities of calibrator) and was assumed as 1. The relative gene expression in B and C (*Y*-axis) was transformed to a log_10_ scale. The values are the means ± SEs of three biological replicates. Data between treatments (112-2-treatment vs. 112-2-CK and JJJD- treatment vs. JJJD-CK) were analyzed by one-way ANOVA and ^*^ denotes statistical significance at *p* < 0.05.

A qRT-PCR assay was completed to analyze the *CmSGT1* expression patterns in PM-resistant inbred line “112-2” and PM-susceptible cultivar “JJJD” treated with PM, H_2_O_2_, SA, abscisic acid (ABA), Eth, and MeJA (Figure 2). The expression data were normalized against that of the β-*actin* gene and were recorded relative to the *CmSGT1* transcript level in the water-sprayed control “JJJD” plants at 0 hour post-inoculation (hpi).

The *CmSGT1* expression level in “112-2” plants were upregulated by PM (except at 24 h), H_2_O_2_, and SA treatments, with the PM-induced expression level at 3 hpi upregulated by 6.62-fold. In contrast, the ABA treatment essentially had no effect. The expression of *CmSGT1* in “JJJD” plants was inhibited by H_2_O_2_, SA, and ABA (except at 24 h) treatments, with irregular expression-level changes induced by PM. After the Eth treatment, the *CmSGT1* expression level was significantly higher in the “112-2” and “JJJD” seedlings than in the water-treatment control (CK) seedlings (except at 48 h), with peak levels occurring at 24 h (i.e., 10.2-fold and 4.5-fold increases in “112-2” and “JJJD” seedlings, respectively). In response to the MeJA treatment, *CmSGT1* expression levels were higher in “112-2” (except at 48 h) and “JJJD” (except at 3 h) seedlings than in CK seedlings. The results indicated that SA, and H_2_O_2_ upregulated *CmSGT1* expression in inbred line “112-2” (PM-resistant material), but downregulated *CmSGT1* expression in cultivar “JJJD” (PM-sensitive material). Moreover, Eth and MeJA induced *CmSGT1* expression regardless of PM susceptibility.

### Improved PM Resistance of *CmSGT1*-Overexpressing *N. benthamiana* Plants

**FIGURE 3:**
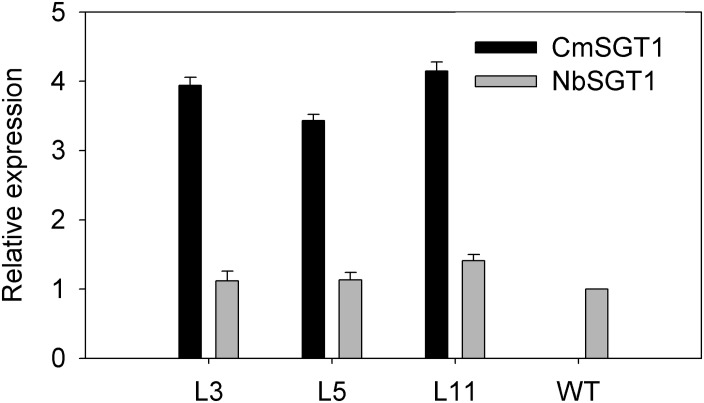
Relative expression of *CmSGT1* and *NbSGT1* in transgenic or wild-type plants under normal growth conditions. *N. benthamiana* encodes *NbSGT1* isoform. L3, L5, and L11 are three independent transgenic lines that overexpress *CmSGT1*. Three biological triplicates were averaged and Bars indicate standard error of the mean. Data (*NbSGT1* expression in transgenic lines vs. *NbSGT1* expression in wild-type plants) were analyzed by one-way ANOVA and unmarked ^*^ denotes no statistical differences at *p* < 0.05.

Firstly, transcript level of the high homology (*NbSGT1*) modulated by the overexpression of *CmSGT1* under normal conditions was determined by qRT-PCR. Compared to wild type (WT) plants, the expression of the homologous genes was not basically altered in transgenic plants when grown in normal condition (Figure 3), indicating that overexpression of pumpkin *CmSGT1* gene has no obvious effect on *NbSGT1* transcript in *N. benthamiana*. The *CmSGT1* gene was not detected in WT plants.

**FIGURE 4:**
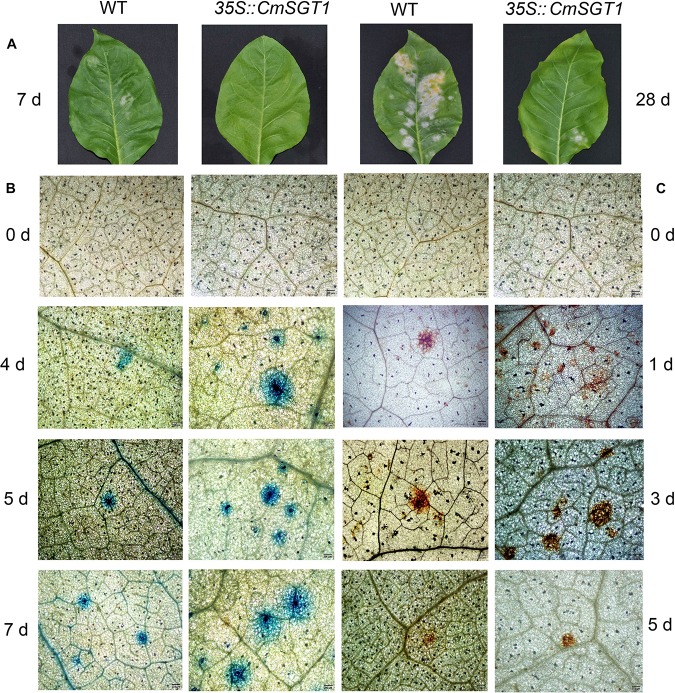
The pathogenic symptoms, trypan blue and DAB staining of *N. benthamiana* leaves treated with powdery mildew. The pathogenic symptoms of transgenic *N. benthamiana* and WT at 7 and 28 days post-inoculation **(A)**; trypan blue staining was performed to visualize cell death **(B)**; DAB staining was performed to visualize H_2_O_2_ accumulation **(C)**. Scale bars = 200 μm.

The disease severity at 10 days post-inoculation (dpi) was 78% lower for the transgenic plants than for the CK plants (Table 1). Powdery mildew symptoms were detectable on infected WT seedlings at 7 dpi, and the infected spots were chlorotic at 28 dpi. In contrast, the PM symptoms were undetectable and relatively slight at 7 and 28 dpi, respectively, in the *CmSGT1*-overexpressing transgenic plants (Figure 4A). A comparison of the leaves of PM-infected WT and transgenic plants revealed more blue spots displayed by the trypan blue staining, on the transgenic leaves than on the WT leaves at 4 dpi, although blue spots were developing on the WT leaves. The blue spots on the transgenic leaves expanded at 5 and 7 dpi, and were bigger than those of WT leaves, implying that the overexpression of *CmSGT1* in transgenic plants accelerated cell death following a PM infection (Figure 4B). Moreover, there were more brown spots manifested by the DAB staining, on the transgenic leaves than on the WT leaves at 1 dpi. These brown spots reflected the accumulation of H_2_O_2_, and were darker brown and larger at 3 dpi. At 5 dpi, the brown spots lightened in the infected plants, but were more intense in the transgenic plants than the spots in the WT plants. The results indicated that the overexpression of *CmSGT1* promoted the accumulation of H_2_O_2_ in transgenic plants infected with PM (Figure 4C).

**TABLE 1:** Disease severity of leaves of *N. benthamiana* seedlings infected with powdery mildew.

**Materials**	**Disease severity (*in vitro* leaf)**
L3	7.90 ± 1.23
L5	8.60 ± 1.31
L11	9.00 ± 1.01
WT	38.80 ± 2.41^*^

Data are mean values (±SD) of at least three independent experiments. “^*^” indicates significantly different values between treatments (*p* < 0.05). L3, L5, and L11 are three independent transgenic lines that overexpress *CmSGT1*.

### Expression of Signal-Related Genes in Transgenic *N. benthamiana* Plants

**FIGURE 5:**
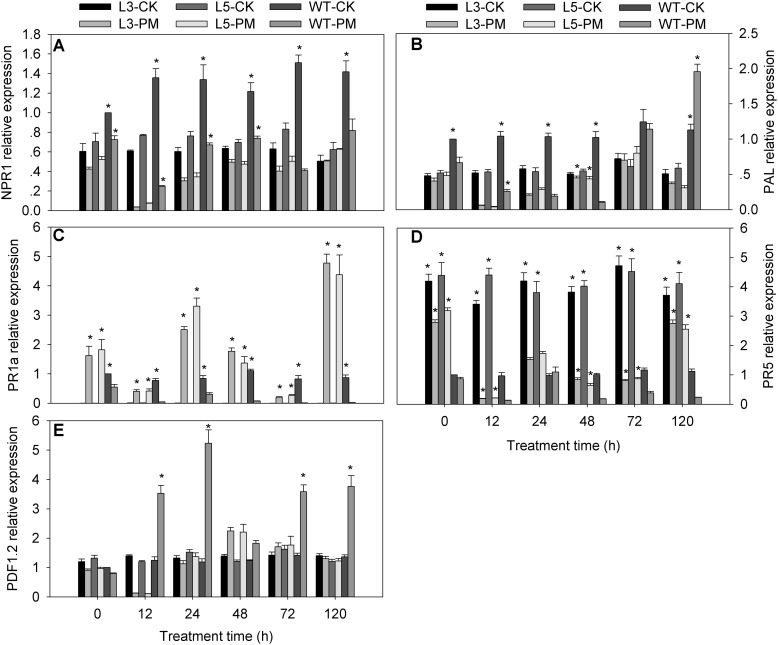
Expression of signal-related genes in transgenic and wild-type plants treated with powdery mildew. The samples of two genetically modified *N. benthamiana* lines were used to analyze the genes expression by qRT-PCR. **(A)** *NtNPR1*; **(B)** *NtPAL*; **(C)** *NtPR1a*; **(D)** *NtPR5*; **(E)** *NtPDF1.2*. L3/5-CK represents transgenic *N. benthamiana* L3 or L5 line grown under normal conditions; L3/5-PM represents transgenic *N. benthamiana* L3 or L5 line infected with powdery mildew; WT-CK represents wild-type plants grown under normal conditions; WT-PM represents wild-type plants infected with powdery mildew. *N. benthamiana NtEF1-α* gene (AF120093) was used as an internal control for normalization of different cDNA samples. The expression levels of signal-related genes in wild-type plants at 0 h were used as control (quantities of calibrator) and were assumed as 1. Three biological triplicates per line were averaged and Bars indicate standard error of the mean. Data between transgenic plants and WT plants (L3/5-PM vs. WT-PM and L3/5-CK vs. WT-CK) were analyzed by one-way ANOVA and “^*^” denotes statistical significance at *p* < 0.05.

To investigate the signal transduction pathways affected by CmSGT1 during plant defense responses to PM, the expression levels of five signaling-associated genes (*NPR1*, *PR5*, *PR1a*, *PAL*, and *PDF1.2*) in the SA, JA, and ET signal transduction pathways were analyzed by qRT-PCR for the *CmSGT1*-overexpressing transgenic and WT *N. benthamiana* plants, with water-treated WT plants at 0 hpi serving as the control samples (Figure 5). The *NPR1*, *PAL* (except at 120 h), and *PR5* expression levels were lower in the transgenic and WT plants infected with PM than in the CK plants, implying that PM inhibited the expression of these genes. The *PR1a* expression level was higher in the PM-infected transgenic plants than in the CK plants (i.e., 68.3-fold at 120 h), whereas the *PR1a* level was lower in the PM-infected WT plants than in the CK plants. The *PDF1.2* expression levels in the PM-infected transgenic plants were lower at 12 hpi and higher at 48 hpi than in the CK plants, with no differences thereafter. Thus, in response to the PM infection, the *NPR1*, *PAL* (except at 24 and 48 h), and *PDF1.2* (except at 48 h) expression levels were significantly lower in the transgenic plants than in the WT plants, whereas the opposite pattern was observed for the *PR1a*, and *PR5* expression levels. These results implied that the overexpression of *CmSGT1* in *N. benthamiana* upregulates the expression of *PR1a*, and *PR5*, which are involved in SA defense signal transduction. Furthermore, the increased PM resistance of the transgenic *N. benthamiana* plants appears to be related to the upregulated expression of these genes.

### Compromised Resistance of Transgenic *N. benthamiana* Plants to Bacterial Diseases

**FIGURE 6:**
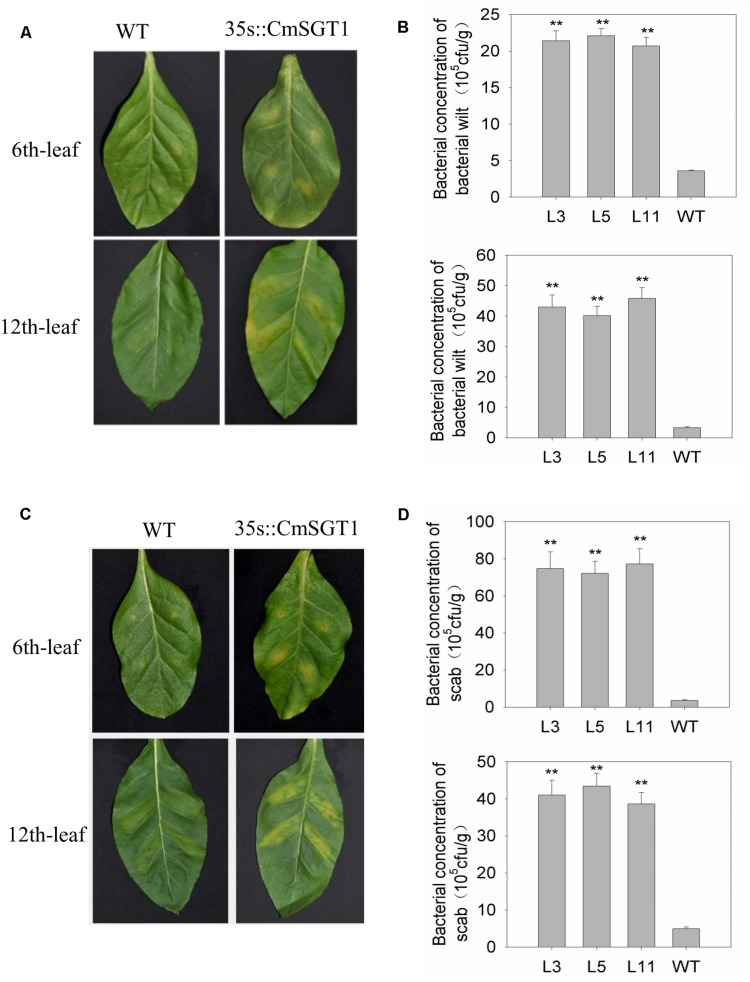
The resistance of *CmSGT1* in transgenic and wild-type plants to *N. benthamiana* bacterial wilt and scab. **(A)** pathogens symptoms of the 6th-upper and 12th-upper leaf injection sites was injected with bacterial wilt bacteria with a needle-removed syringe. **(B)** concentration bacteria of the 6th-upper and 12th-upper leaf injection sites was injected with bacterial wilt bacteria with a needle-removed syringe. **(C)** pathogens symptoms of the 6th-upper and 12th-upper leaf injection sites was injected with scab bacteria with a needle-removed syringe. **(D)** concentration bacteria of the 6th-upper and 12th-upper leaf injection sites was injected with scab bacteria with a needle-removed syringe. “^∗∗^” denotes significant differences between WT and transgenic plants at *p* < 0.01.

To analyze the effect of *CmSGT1* on other plant diseases, two common bacterial pathogens causing bacterial wilt (*Ralstonia solanacearum*) and scab (*Xanthomonas euvesicatoria*) were injected into *N. benthamiana* plants (Figure 6). At 6 days after inoculation with *R. solanacearum* and *X. euvesicatoria*, the chlorosis and yellowing of the sixth leaf veins was greater for transgenic *N. benthamiana* plants than for the WT plants. Additionally, there were 5.94- and 21.1-times more the concentration of bacterial wilt and scab bacteria, respectively, in the transgenic plants than in the WT plants. Yellowing was also observed between the veins of the 12th leaf in transgenic *N. benthamiana* plants, and there were 13.3- and 8.28-times more *R. solanacearum* and *X. euvesicatoria* bacteria, respectively, in the transgenic plants than in the WT plants. These observations suggested that the overexpression of *CmSGT1* in *N. benthamiana* decreases the resistance to bacterial wilt and scab.

## Discussion

In this study, we isolated a novel pumpkin *SGT1* gene, which was designated as *CmSGT1*. The predicted amino acid sequence was 89 and 82% identical to the NbSGT1.1 and CsSGT1 sequences, respectively. In *A. thaliana* and *N. benthamiana*, SGT1 encodes three functional domains (TPR, CS, and SGS) that are essential for SGT1 activity (Peart et al., 2002; Noël et al., 2007). In this study, the CmSGT1-GFP fusion protein was localized to the nucleus in *A. thaliana* protoplasts, which was inconsistent with the results of earlier studies involving SGT1 in other plant species (Xing et al., 2013; Liu et al., 2016). This inconsistency may be related with low identity (*Haynaldia villosa* HvSGT1, 64.0% identity; pepper CaSGT1, 63.7% identity) or the number of transformed cells examined. *Arabidopsis SGT1b* fused to Cerulean localized to the cytosol, but could be seen in nuclei of 25% of 55 transformed cells examined (Noël et al.,2007), and translocation of the SGT1/SRC2-1 complex from the plasma membrane and cytoplasm to the nuclei is required in pepper upon the inoculation of *Phytophthora capsici* (Liu et al., 2016), suggesting the movement of SGT1 between the cytosol and nucleus.

The interplay among complex signaling networks, including various pathways regulated by phytohormones, such as SA, JA, ethylene (ET) and ABA, considerably influences plant resistance to diseases. An earlier investigation proved that *HvSGT1* expression levels substantially increase following treatments with H_2_O_2_ and MeJA, slightly increase following exposure to ET or ABA, and are unchanged in response to SA (Xing et al., 2013). In the current study, SA and H_2_O_2_ treatments considerably upregulated *CmSGT1* expression in the PM-resistant inbred line “112-2”, but downregulated *CmSGT1* expression in the PM-susceptible material. The expression of *CmSGT1* in both materials was upregulated by Eth and MeJA treatments. Recent reports indicated that *SGT1* expression may be induced by phytopathogens, such as *Blumeria graminis* in wheat and *P. capsici* in pepper (Xing et al., 2013; Liu et al., 2016). In this study, the *CmSGT1* expression in inbred line “112-2” was induced by PM. Additionally, the overexpression of *CmSGT1* in transgenic *N. benthamiana* plants decreased the disease index by 78%, accelerated cell necrosis, and increased the accumulation of H_2_O_2_. These results indicated that the PM resistance of the transgenic *N. benthamiana* plants was enhanced, likely because of the changes to the HR-related cell necrosis and H_2_O_2_ accumulation. Our findings are consistent with the results of an earlier study that revealed that H_2_O_2_ accumulation and the subsequent cell death usually lead to the resistance to diseases caused by biotrophic pathogens (Li et al., 2011). Moreover, SGT1 helps mediate cell death during compatible and incompatible plant–pathogen interactions, suggesting that SGT1 is an essential component of common signaling pathways responsible for cell death (Cuzick et al., 2009; Uppalapati et al., 2010; Wang et al., 2010). The silencing of the pepper *SGT1* gene adversely affects HR-related cell death, prevents H_2_O_2_ accumulation, and downregulates HR-related and SA/JA-dependent marker gene expression levels, and influences the PcINF1/SRC2-1-induced pepper defense response by SGT1 interacting with SRC2-1 (Liu et al., 2016).

The *PDF1.2* gene is important for the JA/ET-dependent signaling pathway. The disease-associated *NPR1* gene (non-expresser of PR1) affects various disease-resistance signal transduction pathways, and encodes one of the important transcription factors downstream of SA. The SA-dependent disease-resistance signal transduction pathway can be divided into NPR1-dependent and NPR1-independent transduction pathways (Gao and Zhang, 2012). The *PAL*, *PR1a*, and *PR5* expression levels are markers of the SA signaling pathway. In wheat, HvSGT1 activates PM resistance mechanisms through JA-dependent defense pathways and suppresses the activities of SA-dependent defense pathways (Xing et al., 2013). In the current study, following a PM infection, the *NPR1*, *PAL*, and *PDF1.2* expression levels were lower in the transgenic plants than in the WT plants, whereas the opposite pattern was observed for the *PR1a* and *PR5* expression levels. This suggests that in the SA pathway, the transactivation of *NPR1* is unaffected by CmSGT1, whereas the transactivation of *PR1a*, and *PR5* is dependent on CmSGT1. Additionally, CmSGT1 does not directly affect the JA/ET-dependent defense pathway to regulate *PDF1.2* expression. We propose that *CmSGT1* activates stress-resistance mechanisms through SA-dependent defense pathways without inducing *NPR1* and *PAL* expression levels, but suppresses the activities of JA/ET-dependent defense pathways. Therefore, we speculate that CmSGT1 positively regulates the H_2_O_2_ and SA pathways. Moreover, H_2_O_2_ might directly transfer the SA signal to regulate the expression of downstream response genes in the *CmSGT1*-overexpressing transgenic plants infected with PM. In NPR1-dependent SA signal transduction pathways, the activation of *PR* genes needs NPR1 and TGA transcription factor binding to identify its promoters (Fan and Dong, 2002; Spoel and Dong, 2012). The equivalent mutation in NPR1 abolishes its ability to bind SA and promotes SA-induced defense gene expression (Ding et al., 2018). We speculated that whether there is the relation of the downregulation of *NPR1* and SA-mediated transcriptional activation of *PR* genes or not. Notably, the phenotypes and genes in *CmSGT1*-overexpressing transgenic *N. benthamiana* plants infected with *N. benthamiana* PM might not be exactly the same as those regulated by *CmSGT1* in *C. moschata* in response to cucurbit PM. Further studies will be necessary to reveal biological functions for *CmSGT1* in *C. moschata* infected with PM.

Two globally important diseases that affect *N. benthamiana*, bacterial wilt and scab, are caused by the necrotrophic *R. solanacearum* and the hemi-biotrophic *X. euvesicatoria*, respectively. There are differences in the resistance mechanisms and patterns of fungal development for biothophic (*P. xanthii*) and necrotrophic (*R. solanacearum*) pathogens. Recent studies confirmed that SGT1 promotes the resistance to biotrophic pathogens, while suppressing the resistance to necrotrophic and hemibiotrophic pathogens. Silencing of *SGT1* compromised resistance to the barley and *H. villosa* biothoph *B. graminis* (Shen et al., 2003; Xing et al., 2013) and the wheat biotroph *Puccinia striiformis* (Scofield et al., 2005), while enhancing the resistance of *N. benthamiana* to the necrotrophic pathogen *Botrytis cinerea* (El Oirdi and Bouarab, 2007). A recent study involving *N. benthamiana* indicated silencing of *NbSGT1* compromised the protective effect of systemic acquired resistance-induced plants on neighboring plants against bacteria wilt caused by *R. solanacearum* (Cheol Song et al., 2016). In the current study, the chlorosis and yellowing of the infection sites on leaves were greater in transgenic *N. benthamiana* plants than in WT plants at 6 dpi. Additionally, there were substantially more concentration of bacterial wilt and scab bacteria in the transgenic plants than in the WT plants, indicating that the overexpression of *CmSGT1* in *N. benthamiana* adversely affects the resistance to bacterial wilt and scab, which differs from the effects of *CmSGT1* overexpression on the resistance to PM. Consequently, SGT1 may function differently depending on the plant–pathogen combinations with diverse effectors and R proteins.

In conclusions, the results of this study indicate that the overexpression of *CmSGT1* may increase the PM resistance, but decrease bacterial wilt and scab resistance, in transgenic *N. benthamiana* plants. Additionally, *CmSGT1* overexpression may improve PM resistance by enhancing HR-related cell death and H_2_O_2_ accumulation and upregulating the expression of SA-mediated defense-response genes. Further analyses of the *CmSGT1* gene may be useful for characterizing biotic stress signaling pathways and for the genetic engineering of novel pumpkin cultivars. The results described herein may be relevant for future biotechnology-based investigations and the molecular breeding of pumpkins and related plant species.

## Materials and Methods

### Plant Materials and Treatments

Pumpkin (*C*. *moschata*) inbred line “112-2” and cultivar “Jiujiangjiaoding” (abbreviated “JJJD”), which are resistant and susceptible to PM, respectively, were provided by the Henan Institute of Science and Technology, Xinxiang, Henan, China (Zhou et al., 2010). Pumpkin seeds were germinated and the resulting seedlings were grown as previously described (Guo et al., 2018). Seedlings at the three-leaf stage were treated as follows. The PM infection was initiated as described by Guo et al. (2018). Specifically, conidia were collected from the leaves of pumpkin plants naturally infected with PM in a local greenhouse. Seedlings were sprayed with a freshly prepared spore suspension (10^6^ spores/ml) or an exogenous signaling molecule or hormone, including 1.5 mM H_2_O_2_, 100 μM SA, 100 μM ABA, 100 μM MeJA, 0.5 g/L Eth. Moreover, water alone was used for the control treatment (CK). The treated seedlings were maintained in a growth chamber with a 15-h light (28°C)/9-h dark (18°C) cycle (5,500 lux light intensity) and harvested after 0, 3, 6, 12, 24, and 48 h to examine the *CmSGT1* expression pattern. At each time point, two young leaves were collected from the upper parts of four seedlings (i.e., one sample), wrapped in foil, frozen in liquid nitrogen, and stored at −80°C. The treatments were arranged in a randomized complete block design, with three biological replicates.

### Isolation of *CmSGT1* cDNA Clone and Sequence Analysis

The *SGT1* homolog expressed sequence tag (GenBank accession number SRR5369792) was identified in a PM-resistant pumpkin seedling transcriptome by Guo et al. (2018). The full-length ORF of the *SGT1* homolog was obtained with the cDNA fragment of this homolog used as a probe, as previously described (Guo et al., 2014). The theoretical molecular weight (Mw) and isoelectric point (pI) were calculated with the ExPASy Compute pI/Mw tool (Bjellqvist et al., 1993). Sequence data were analyzed with the ClustalW program (Thompson et al., 1994). The NCBI databases were screened for homologous sequences with the default parameters of the BLAST program^2^ (Altschul et al., 1997).

### Subcellular Localization Analysis of CmSGT1

The *CmSGT1* ORF (without the termination codon) was ligated into the pBI221-GFP vector for the subsequent production of a green fluorescent protein (GFP)-tagged CmSGT1 fusion protein. Polyethylene glycol was used during the transformation of *A. thaliana* protoplasts with the recombinant plasmid (Lee et al., 2013). The subcellular localization of CmSGT1 was determined based on the GFP signal, which was detected with the confocal fluorescence microscope (UltraVIEW VoX, Olympus, Japan) under excitation wavelength 488 nm and captured light wavelength range 448–508 nm. The cell membrane and nucleus were stained with 1,1′-dioctadecyl-3,3,3′,3′-tetramethylindocarbocyanine perchlorate (Dil) and 2-(4-Amidinophenyl)-6-indolecarbamidine dihydrochloride (DAPI), respectively.

### Generation of *CmSGT1*-Overexpressing Transgenic *N. benthamiana* Plants

Pumpkin are known to be one of the plants most refractory for transformation. To date, only two reports on transformation in *C. moschata* existed using a combined method of vacuum infiltration and *Agrobacterium* infection (Nanasato et al., 2011; Nanasato and Tabei, 2015). So, we choose a heterologous overexpression assay in *N. benthamiana* instead of generating *C. moschata* transgenic plants. Forward and reverse primers with an added *Bam*H I site and *Kpn* I site, respectively, were used to amplify *CmSGT1*. The amplified sequence was then inserted into the pVBG2307 vector for the subsequent expression of *CmSGT1* under the control of the 35S cauliflower mosaic virus (CaMV) promoter. The recombinant plasmid was introduced into *Agrobacterium tumefaciens* GV3101 cells as previously described (Guo et al., 2014). The resulting *A. tumefaciens* cells were used to transform *N. benthamiana* plants according to a previously described leaf disk method (Li et al., 2012). The transgenic *N. benthamiana* plants were confirmed by examining the segregation ratio of the kanamycin selectable marker and by PCR analysis of *NPTII* and *CmSGT1*. T2 lines that produced 100% kanamycin-resistant plants in the T3 generation were considered as homozygous transformants. In each experiment, T2 generations of homozygous transgenic lines (L3, L5 and L11) were selected for further analysis. Similar phenotypes and results used for this study were observed in more than three independent lines of transgenic plants.

### Primer Design

Details regarding all of the primers designed and used in this study are provided in Supplementary Table S1.

### Analysis of Transgenic *N. benthamiana* Plants Infected With Powdery Mildew

Conidia were collected from *N. benthamiana* leaves naturally infected with *Erysiphe cichoracearum* DC., which causes PM. Transgenic *N. benthamiana* seedlings at the five-leaf stage were used for phenotypic analyses. Specifically, the petiole of the second leaf from the top of the seedlings was wrapped with cotton moistened with water. The leaf was placed on a porcelain tray containing filter paper, after which it was sprayed with a spore suspension (10^6^ spores/ml). The tray was then covered with film to maintain humidity. At 10 dpi, disease severity of leaves *in vitro* was calculated as [(5*A* + 4*B*+ 3*C*+ 2*D*+ *E*)/5*F*] × 100 according to Ishii et al. (2001). Additionally, the first fully expanded true leaf of the transgenic and WT plants were sprayed with the above-mentioned spore suspension and sampled after 0, 12, 24, 48, 72, and 120 hpi, frozen in liquid nitrogen, stored at −80°C and used for extraction of total RNA. On the other hand, these leaves were harvested symmetrically along the sides of the main vein after 0, 1, 3, 4, 5, and 7 days to examine of cell death and H_2_O_2_ accumulation. The treatments were arranged in a randomized complete block design with three replicates.

Leaves from the transgenic *N. benthamiana* seedlings were stained with 3, 3′-diaminobenzidine (DAB) and trypan blue staining as previously described (Choi et al., 2012) to analyze H_2_O_2_ accumulation and cell death. For DAB staining, whole leaves were immersed in DAB solution (1 mg/ml, pH 5.7) and incubated overnight (almost 8 h) in darkness. Leaves were then de-stained three times with 95% ethanol. Regarding trypan blue staining, whole leaves were boiled in a lactophenol-ethanol trypan blue solution (10 ml lactic acid, 10 ml glycerol, 10 g phenol, 30 ml absolute ethanol, and 10 mg trypan blue dissolved in 10 ml distilled water) for 10 min and then maintained at room temperature overnight. Leaves were de-stained with 2.5 g/ml chloral hydrate in distilled water.

### Analysis of Transgenic *N. benthamiana* Plants Infected With Bacterial Wilt and Scab

The sixth and twelfth leaf from the top of transgenic *N. benthamiana* seedlings were inoculated with bacterial solutions (10^8^ cfu/ml). Specifically, the bacterial solutions were injected into the underside of leaves between the lateral veins with a syringe lacking a needle. Each leaf was inoculated in four places. The petiole of leaves placed on a porcelain plate was wrapped with water-saturated degreased cotton, after which the plate was covered with film to maintain humidity and then incubated at 28°C with a 16-h light/8-h dark cycle. Water was periodically added to the cotton to maintain an appropriate moisture level. Experiments were done in triplicate for each line. After a 6-day incubation, the concentration of bacteria at the injection sites was determined as follows. The injection sites were sampled with a circular perforator (1 cm diameter) and ground in aseptic water (0.1 g added to 0.9 ml water). The ground material was serially diluted to produce the 10^2^-fold, 10^3^-fold, and 10^4^-fold diluents. A 0.1-ml aliquot of the 10^3^-fold, 10^4^-fold, and 10^5^-fold diluents were added to a petri dish containing NA medium. The medium was incubated upside down at 28°C. Plates with 30–300 colonies were considered ideal. The bacterial concentration was calculated with the following formula: colony average × 10 × dilution/g.

### Quantitative Real-Time PCR Analysis

The RNA extraction, first-strand cDNA synthesis and quantitative real-time (qRT-PCR) were completed as described by Guo et al. (2015). Relative gene expression levels were determined with the 2^–ΔΔ*CT*^ method. Total RNA was extracted from the leaves of pumpkin seedlings treated with various stresses or distilled water for 0, 3, 6, 12, 24, or 48 h as described above. The β-*actin* gene was used as an internal control, because it was confirmed as a suitable reference gene for normalizing of gene expression levels in pumpkin (Wu and Cao, 2010). On the other hand, total RNA was extracted from *CmSGT1*-overexpressing transgenic and WT *N. benthamiana* seedlings to examine the expression of five hormone-related genes (*NtNPR1*, *NtPR1a*, *NtPR5*, *NtPDF1.2*, and *NtPAL*) and *N. benthamiana* isoform (*NbSGT1*). The *N. benthamiana NtEF1-α* gene (AF120093) was included in the assays as an internal control.

### Statistical Analyses

Values were expressed as the mean ± standard error of three independent determinations. Data were compared by analysis of variance (ANOVA) using a one-way ANOVA, and differences between WT and transgenic plants were tested by a *post hoc* comparison test (Student–Newman–Keuls) at *p* < 0.05 with SPSS 19.0 for Windows (SPSS Inc., Chicago, IL, United States).

## Data Availability

The datasets generated for this study can be found in NCBI, MH105820.

## Author Contributions

W-LG and Y-YG conceived and designed the experiments. W-LG and B-HC performed the experiments. J-YM and H-LY analyzed the data. Y-LW, J-GZ, and X-ZL contributed reagents, materials, and analysis tools. W-LG wrote the manuscript.

## Conflict of Interest Statement

The authors declare that the research was conducted in the absence of any commercial or financial relationships that could be construed as a potential conflict of interest.

## Footnotes

1 http://www.fao.org/faostat/en/#data/QC

2 http://www.ncbi.nlm.nih.gov/blast

## Supplementary Material

FIGURE S1 Amino acid sequences alignment of pumpkin CmSGT1 with others. The three conserved domains (TPR, CS, and SGS) are shown by thin underlines. The genes included are CmSGT1 (*Cucumis melo* L., XP_008439299.1) and CsSGT1 (*Cucumis sativus* L., XP_004140745.1) AtSGT1A (*Arabidopsis*, AT4G23570.3) and AtSGT1B (*Arabidopsis*, AT4G11260.1), NbSGT1.1 (*N. benthamiana*, AF516180) and NbSGT1.2 (*N. benthamiana*, AF516181).

TABLE S1 Primers used in this investigation.
