# Supplementary material for: Improved Powdery Mildew Resistance of Transgenic Nicotiana benthamiana Overexpressing the Cucurbita moschata CmSGT1 Gene
Source: Front Plant Sci. 2019 Jul 25;10:955. doi: 10.3389/fpls.2019.00955 (PMC6670833; doi:10.3389/fpls.2019.00955)
Supplement: FIGURE S1 — Amino acid sequences alignment of pumpkin CmSGT1 with others. The three conserved domains (TPR, CS, and SGS) are shown by thin underlines. The genes included are CmSGT1 (Cucumis melo L., XP_008439299.1) and CsSGT1 (Cucumis sativus L., XP_004140745.1) AtSGT1A (Arabidopsis, AT4G23570.3) and AtSGT1B (Arabidopsis, AT4G11260.1), NbSGT1.1 (N. benthamiana, AF516180) and NbSGT1.2 (N. benthamiana, AF516181). [file Image_1.pdf]

```
CsSGT1      MASDLEAKAKEAFIDDHFELSVDLYTQAIALSPKKSELYVDRAQANIKLGHYT.ETVADA   59
CmSGT1      MASDLEAKAKEAFIDDHFELSVDLYTQAIALSPKKSELYVDRAQANIKLGHYT.EAVADA   59
NbSGT1_1    MASDLEIRAKEAFIDDHFELAVDLYTQAIAMTPKNAELFADRAQANIKLNYFT.EAVVDA   59
NbSGT1_2    MASDLEIRAKEAFIDDHFELAVDLYTQAIAMTPKNAELFADRAQANIKLNYFT.EAVVDA   59
AtSGT1a     MAKELADKAKEAFVDDDFDVAVDLYSKAIDLDPNCAEFFADRAQAYIKLESFTAEAVADA   60
AtSGT1b     MAKELAEKAKEAFLDDDFDVAVDLYSKAIDLDPNCAAFFADRAQANIKIDNFT.EAVVDA   59
CmSGT1      MASDLEARAKEAFIDDHFELSVELYTQAIALSPKKAELYIDRAQANIKLGHYT.EAVADA   59
Consensus   ma  l   akeaf dd f    v ly  ai   p      iraqa ik     t e v da
                                                 TPR

CsSGT1      NKAIELDPSNSKAYLRKGTACMKLEEYQTAKAALETGSALAPGDSRFTNLIKECEKLIAE  119
CmSGT1      NKAIELDPSNSKAYLRKGTACLKLEEYQTAKVALETGSALASGDSRFTNLIKECEKLIAE  119
NbSGT1_1    NKAIELDPSMSKAYLRKGLACMKLEEYQTAKAALETGASLAPAESRFTKLIKECDERIAE  119
NbSGT1_2    NKAIELDPSMSKAYLRKGLACMKLEEYQTAKAALETGASLAPAESRFTKLIKECDERIAE  119
AtSGT1a     NKAIELDPSLTKAYLRKGTACMKLEEYRTAKTALEKGASITPSESKFKKLIDECNFLITE  120
AtSGT1b     NKAIELEPTLAKAYLRKGTACMKLEEYSTAKAALEKGASVAPNEPKFKKMIDECDLRIAE  119
CmSGT1      NKAIELDPSNSKAYLRRGTACLKLEEYQTAKVALETGSAMASGDSRFSSLIKECEKLIAG  119
Consensus   nkaiel p   kaylr g ac kleey tak ale g           f   i ec   i
                                                    TPR

CsSGT1      EMGDLTQESVEND....VQETVKSSADIVPVSDQLHQATI.......EVKPKFRHEYYQK  168
CmSGT1      EMGDLTQESVENN....VQETVKSSADIVPVSDQLHHATI.......EVKPKFRHEYYQK  168
NbSGT1_1    EAGELPNQSVDKTSGNVVAPPASESLGNVAVAPKDAQPTVNLSYQGSAARPKYRHEFYQK  179
NbSGT1_2    EAGELPNQSVDKTSGNVVAPPASESLGNVAVAPKDAQPTVNLSYQGSAARPKYRHEFYQK  179
AtSGT1a     EEKDLVQP.........VPSTLPSSVTAPPVSELDVTPT.........AK..YRHEYYQK  160
AtSGT1b     EEKDLVQP.........MPPSLPSSSTTPLATEADAPPVP...IPAAPAKPMFRHEFYQK  167
CmSGT1      EMGDLPQESVEET....VQEPVLPTNNVVPVSDQLHQATV.......EAKPKFRHEFYQK  168
Consensus   e   l                                                rhe yqk

CsSGT1      PEEVVVTIFAKGIPAENVAVQFGEQILSVTIDLPGEDAYCFQARLFGKIIREKCKFFVLS  228
CmSGT1      PEEVVVTIFAKGIPAENVAVQFGEQILSVTIDLPGEDAYRFQPRLFGKIIREKCKFFVLS  228
NbSGT1_1    PEEVVVTIFAKGIPAKNVIVDFGEQILSVSIDVPGDETYSFQPRLFGKITPAKCRYEVMS  239
NbSGT1_2    PEEVVVTIFAKGIPAKNVIVDFGEQILSVSIDVPGDETYSFQPRLFGKITPAKCRYEVMS  239
AtSGT1a     PEEVVVTVFAKGIPKQNVNIDFGEQILSVVIEVPGEDAYYLQPRLFGKIIPDKCKYEVLS  220
AtSGT1b     PEEAVVTIFAKKVPKENVTVEFGEQILSVVIDVAGEEAYHLQPRLFGKIIPEKCRFEVLS  227
CmSGT1      PEEVVVTIFAKGIPAESVAVEFGEQILSVTINLPDEDAYRFQTRLFGKIIRGKCKFLVLS  228
Consensus   pee vvt fak  p    v  fgeqilsv i        y  q rlfgki    kc   v s
                                             CS

CsSGT1      TKIEIRLVKAEQIHWTSLEFSKENRIIPSISVPSSGSQRPSYPSSKPRR.DWDKIEAEVK  287
CmSGT1      TKIEIRLVKAEQIHWTSLEFSKENRIIQSINVPSSGSQKPSYPSSKPRR.DWDKIEAEVK  287
NbSGT1_1    TKIEIRLAKAEPLHWTSLEYTRESAVVQRPNVS.SDAPRPSYPSSKLRHTDWDKLEAEVK  298
NbSGT1_2    TKIEIRLAKAEPLHWTSLEYTRESAVVQRPNVS.SDAPRPSYPSSKLRHTDWDKLEAEVK  298
AtSGT1a     TKIEICLAKADIITWASLEHGKGPAVLPKPNVSSEVSQRPAYPSSKKVK.DWDKLEAEVK  279
AtSGT1b     TKVEIRLAKAEIITWASLEYGKGQSVLPKPNVSSALSQRPVYPSSKPAK.DWDKLEAEVK  286
CmSGT1      TKIEIRLAKAEPITWTSFEFKKDNRITQSISVPASGSQRPPYPSSKPKR.DWDKIEAEVK  287
Consensus   tk ei l ka    w s e             v       p ypssk    dwdk eaevk

CsSGT1      KEEKDEKLEGDAALNKFFRDIYGDADEDTKRAMEKSFVESNGTVLSTNWKEVGSKKVEGS  347
CmSGT1      KEEKDEKLDGDAALNKFFRDIYKDADEDTRRAMQKSFVESNGTVLSTNWKEVGSKKVEGS  347
NbSGT1_1    KEEKDEKLDGDAALNKFFRDIYKDADEDTRRAMMKSFVESNGTVLSTNWKEVGAKKVEGS  358
NbSGT1_2    KEEKDEKLDGDAALNKFFRDIYKDADEDTRRAMMKSFVESNGTVLSTNWKEVGAKKVEGS  358
AtSGT1a     KQEKDEKLEGDAALNKFFREIYQNADEDMRRAMSKSFVESNGTVLSTNWQEVGTKTIEST  339
AtSGT1b     KQEKDEKLDGDAAMNKFFSDIYSSADEDMRRAMNKSFAESNGTVLSTNWKEVGTKKVEST  346
CmSGT1      KEEKDEKLDGDAALNKFFRDIYKDADEDTRRAMQKSFVESNGTVLSTNWKEVGTKKVEGS  347
Consensus   k ekdekl gdaa nkff  iy  aded  ram ksf esngtvlstnw evg k  e

                                            SGS
CsSGT1      PPDGMELKKW..                                                  357
CmSGT1      PPDGMELKKWL.                                                  358
NbSGT1_1    PPDGMELKKWEI                                                  370
NbSGT1_2    PPDGMELKKWEI                                                  370
AtSGT1a     PPDGMELKKWEI                                                  351
AtSGT1b     PPDGMELKKWEY                                                  358
CmSGT1      PPDGMELKKWEI                                                  359
Consensus   ppdgmelkkw
```
