# Supplementary material for: Improved Powdery Mildew Resistance of Transgenic Nicotiana benthamiana Overexpressing the Cucurbita moschata CmSGT1 Gene
Source: Front Plant Sci. 2019 Jul 25;10:955. doi: 10.3389/fpls.2019.00955 (PMC6670833; doi:10.3389/fpls.2019.00955)
Supplement: TABLE S1 — Primers used in this investigation. [file Table_1.docx]

Table S1 Primers used in this investigation

| Gene | Accession | Primer sequence(5’-3’) |
| --- | --- | --- |
| *CmSGT1* | MH105820 | F: CATCAGTTATCAAGACTTCCAAGTC  R: GAGTTGAAGAAATGGGAGATCTGAT |
| RT-qPCR for *CmSGT1* |  | F: ATTACCCAGAGCATTAGTGTCCC  R: TATCCTCGTCAGCGTCCTTGTAT |
| Over-  expression  vector |  | F:GGGGATCCCATCAGTTATCAAGACTTCCAAGTC (*BamH* I)  R:GGGGTACCGAGTTGAAGAAATGGGAGATCTGAT ( *Kpn* I) |
| *NtNPR1* | U76707 | F: ACATCAGCGGAAGCAGTAG  R: GTCGGCGAAGTAGTCAAAC |
| *NtPR1a* |  | F: CCTCGTACATTCTCATGGTCAAT  R: CCATTGTTACACTGAACCCTAGC |
| *NtPR5* |  | F: CCGAGGTAATTGTGAGACTGGAG  R: CCTGATTGGGTTGATTAAGTGCA |
| *NtPDF1.2* | T04323 | F: GGAAATGGCAAACTCCATGCG  R: ATCCTTCGGTCAGACAAACG |
| *NtPAL* | X95342 | F: GTTATGCTCTTAGAACGTCGCCC  R: CCGTGTAATGCCTTGTTTCTTGA |
| *NtEF1-α* | AF120093 | F: TGTGATGTTTTTGTTCGGTCTTTAA  R: TCAAAAGAAAATGCAGACAGACTCA |
| *NbSGT1* | AF516180 | F: GGTTGTTTGGGAAGATAACACC  R: ATTTCGAGGAAGGATAACTGGG |
| *β-actin*  *NPTII* |  | F: TCTCTATGCCAGTGGTCGTA  R: CCTCAGGACAACGGAATC  F: AGACAATCGGCTGCTCTGAT  R: TCATTTCGAACCCCAGAGTC |
